# 1945–1964 WHO’s Right to Health?

**DOI:** 10.1007/s00048-022-00333-y

**Published:** 2022-05-24

**Authors:** Linda M. Richards

**Affiliations:** Oregon State University, Corvallis, USA

**Keywords:** history of radiation protection, IAEA, WHO, UN, USAEC, UN technical experts, Chisholm, Candau, Pauling, human rights

## Abstract

United States Atomic Energy Commission (USAEC) and UN agencies utilized techniques of power and negotiation to implement radiation exposure regulations. USAEC affiliated scientists’ expertise was cultivated while establishing a radiation protection regime based on classified experiments. World Health Organization (WHO) leadership sought to manifest a human right to health, including a right to protection from radiation contamination. The careers of a few technical experts and interagency UN correspondence shows how American risk models of radiation regulation traveled and ultimately inhibited WHO attempts to frame radiation as a public health threat. The USAEC and the International Atomic Energy Agency (IAEA) navigated WHO’s way of perceiving radiation with technical experts and bureaucratic and legislative means. This paper shows the underpinning at the UN of competing models of radiation regulation, one state centric and the other, an individual right to health. This narrative provides insights into the nature of the UN’s current conceptualization of radiation regulation and argues for further research into UN, radiation, and human rights history.

Scientists’ faith in the “promise of the atom” helped construct the UN global order (Hamblin 2021: 256; Roehrlich 2018). Science diplomacy bloomed in the United Nations, especially among technical experts. Many such technical nuclear experts were shared by the World Health Organization (WHO), United Nations Educational, Scientific and Cultural Organization (UNESCO), Food and Agriculture Organization (FAO) and International Atomic Energy Agency (IAEA) (Mateos & Suárez-Díaz 2021; Sharp 1952: 61; Richards 2014: 75–6; Alexander 1966).^1^ The US Atomic Energy Commission (USAEC) expedited and promoted nuclear relationships and networks with a focus on atomic diplomacy. UN technical experts shared health physics, nuclear science, and technology on local to global scales. These experts intersected with the USAEC, national atomic energy commissions, military and research labs, governments, corporations, the public, and educational institutions. They even trained high school teachers (Westwick 2003; Creager 2013; Krige 2006). One UN expert characterized himself as an “exchange particle.”^2^

Scholars are mapping some of the complex interrelationships that built the world’s nuclear infrastructure (Petryna 2002; Hecht 2006; Kuchinskaya 2014; Rentetzi 2017). This paper relies on James Secord’s concept of knowledge in transit combined with John Krige’s ideas about the circulation of science regulation to try to “see” how radiation health safety traveled (Secord 2004; Krige 2014). While secrecy limits access to sources on nuclear programs, I argue that it is often the case that obscure scientists played a pivotal role, along with bureaucratic and legislative methods, in institutionalizing the USAEC understanding and regulation of radiation health safety at the UN and worldwide. This paper uses documents drawn from USAEC and US university archives to explore how certain US experts cultivated radiation expertise during a period of crafting of radiation regulations into the mid-1950s. I contrast this process with other ways of seeing radiation as a public health threat in the same era. My synthesis of UN documents and secondary literature shows WHO leadership advocated for a right to health that included protection from radiation.

The WHO and the IAEA competed for authority and influence in the sphere of radiation regulation. They each sought jurisdiction over ways of interpreting radiation, as either a threat to the human right to health or a tool for development but American ideas about radiation regulation in the postwar era dominated. This paper shows the USAEC and IAEA used techniques of power and negotiation to design and implement scientific regulatory structures at the UN to the mid 1960s. Two seemingly irreconcilable logics of radiation regulation competed at the UN. One was an American state-centric model, which could be described as a “right to contaminate” in order to produce nuclear weapons and spread benefits from nuclear science. This logic traveled with UN technical experts, predicated on a resolve to expand and export nuclear technology. This interpretation of radiation exposure as allowable up to a certain point contrasted with a conception of an individual right to health; a right to live free from threats of nuclear war or exposure to risk and harm from additional artificial ionizing radiation.

## Cultivating Radiation Expertise

The US origins of standard setting in the field of radiation health safety set the stage for later jurisdictional conflicts over radiation research at the UN. The UN General Assembly in 1946 intended to create global scientific research under their aegis to benefit the world. An October 3, 1946 resolution stated “some research can only be conducted in a rational manner on an international scale.” Science, as the resolution itself asserted, would be better organized in UN research labs, “especially for public health.”^3^ WHO sponsored their first radioisotopes training in 1948.^4^ But radiation research could not really be international. This is because the basis for understanding radiation and its regulation relied on classified human and animal research projects. Much of the data was secret and belonged only to the USAEC (Krige 2006; Brodie 2015; Lindee 1994, 2016). Building trust in the nuclear future required faith in a science that only USAEC-approved eyes could see.

US military and utilitarian uses shaped influential regulatory logic that portrayed radiation contamination as an allowable and worthwhile risk. Secrecy for national security, utilitarian aims and disciplinary boundaries complicated understanding radioactivity (Jolly 2003). Three main US research considerations were laboratory safety for scientists, waging or surviving nuclear war, and finding “fruitful ways to use the unique tools of atomic energy” (USAEC 1951: 23–25). Since radiation can penetrate imperceptibly inside bodies, three approaches evolved to calculate unseen internal radiation exposure: direct, indirect, and computational (Stannard 1988: 14).

The direct approach established a limit for radium 226 exposure by 1941. This was the original informal guideline used for the Manhattan Project. To establish this measure, patients, radiologists, chemists suffering from radium exposure, and ill and dying radium dial painters, were used in experiments that gathered “either breath analysis for radon or gamma ray measurements.” This often took place without their knowledge or consent. Scientists then correlated these measurements with perceived health effects. These studies instrumentalized suffering people as research objects (Rentetzi 2004; Stannard 1990:14–15).

The indirect approach was more vast and complicated. Extensive USAEC funded studies examined and observed Hiroshima and Nagasaki survivors, Marshall Islanders, atomic soldiers, uranium miners, pregnant women, children, unwitting patients and others, sometimes without subjects’ knowledge or consent (Wasserman & Solomon 1982; Lindee 1994, 2016; Mitchell 2017). Doctors and scientists injected patients judged to be terminally ill with radioactive materials and meticulously studied their bodies after death (Fink 1950; Stannard 1988; Welsome 1999; Creager 2013: 260–310). USAEC and US government agencies like the Public Health Service funded scientists and physicians to transform human suffering into data to be used in the development of radiation regulatory regimes.^5^

Health physicists combined the human data with the results from experiments on animals, including rats, rabbits, dogs, pigs, burros, and sheep. Scientists, for example, compared aging and other effects between beagles and humans to establish toxicity ratios (Fink 1950; Stannard 1988; Bolman 2018). The findings could be too restrictive when taken literally. For instance, the toxicity ratio for plutonium “would have closed Los Alamos,” but a scientist applauded how “biological science found a way out.” The retention of plutonium in mice was higher than its absorption by human bodies to justify more relaxed protective measures than the toxicity studies indicated (Stannard 1990:16). Thus, models could be manipulated for utility. Radioecology studies of uptake from the environment were also factored in. Soils, rivers, school children and bones were secretly observed, while authorities near nuclear sites often advertised any publicly known monitoring or radiobiology studies as a guarantee of safety (Pritikin 2020; Walker 2000: 14–18; Stannard 1988, 1990: 20, 29–30).

Eventually, these types of direct and indirect data were embedded in a computational approach to determine exposure limits. Nuclear fission and fusion produce hundreds of individual radionuclides and each decays at differing rates. Scientists needed to factor this into the possible effect on individual organs. This was an overwhelmingly complex challenge, even when focused on only the most dangerous elements. USAEC affiliated scientists and new computational systems took part in this insular process that created these “black-boxed facts.” In this way, radiation harm was studied and interpreted to build a motif of a threshold. In the models they produced, averages and estimates of background radiation from “natural levels” of uranium and cosmic rays served as a proxy for harmlessness (Libby 1955; Latour & Woolgar 1979; Richards 2014; Stannard 1990: 14–20).

USAEC studies like Project Sunshine contributed to radiation safety standards while developing a shared logic of radiation contamination as safe when below certain amounts. Joseph Shirley Butts, a biochemist on a two-year leave from Oregon Agricultural College (OAC, now Oregon State University) from 1952 to 1954, served as Assistant Director of the USAEC’s Biology and Medicine division. In that role, Butts oversaw aspects of the Project Sunshine study. Butts was known for his selflessness as one of the first civilians at the Oregon college to volunteer in WWII, surviving a torpedo attack on his way to be a nutrition officer in England (SCARC 2009; Science 1952: 532).

Butts attended a 1948 USAEC summer training at Oak Ridge, Tennessee to learn how to use radioisotopes as a tool for agriculture. He glued a photo of himself in his lab notebook with his fellow trainees, all grinning on the porch of a military-style barrack named the “Rutherford Hotel.” The USAEC encouraged early researchers of radioisotope tracers like Butts to be ardent boosters for the utopian philosophy later embedded in the Atoms for Peace program. In 1950, 194 scientists and technicians attended a similar four-week course in radiation safety and 25 Universities participated in Oak Ridge’s radioisotope training programs. At Oak Ridge, and at another month-long Brookhaven Lab training, Butts gained access to top secret information. He also secured fellowship opportunities in USAEC Biology and Medicine for OAC students (Creager 2013: 86, 317; Mateos & Suárez-Díaz 2021; USAEC 1951:17; Richards, 2014: 203).^6^

Back on campus, Butts posed for photos in front of a crane installing a huge cyclotron magnet in 1950 that would make isotopes. He stood with his colleagues, including Richard R. Dempster, one of two scientists who came to OAC from Los Alamos in 1944 in order to train more nuclear physicists.^7^ By the early 1950s the USAEC was embedded into academic institutions like Oregon, offering prestige and opportunity along with easy to replicate curriculums (Doel 2003; Hewlett & Holl 1989: 253–6; Westwick, 2003). Scientists who became involved with radiation or radioisotopes inside and outside the US were trained in USAEC-developed and approved courses in health physics (Creager 2013; Santesmases 2006; Krige 2019a, b).^8^ American fellowships in Health Physics from the USAEC required not just loyalty oaths, but an FBI investigation and a USAEC security clearance (USAEC 1951: 35). As Butts found, there were many USAEC federal grants for training and equipment like research reactors with support for studies in various fields, from physics to biology to agriculture to medicine, “with fallout … one of the most significant challenges and boosts that environmental science ever received” (Stannard 1988: 883).

Butts worked on Project Sunshine as a part of a network of scientists around the globe. Scientists collected samples to indicate just how much manmade strontium 90 was already in humans. Later aspects of the study gauged the radiation in the environment. These scientists developed the first “systematic studies of the ecological behavior of Sr 90” caused by nuclear weapons contamination (Eisenbud 1990:78; Creager 2013: 130). Some scientists found wide variations in bones “from one individual to another” while others reported a “marked consistency” of the deposition of strontium over a large number of individuals. Butts reconciled these variations in irregularly collected sample results from human and fetal bones.^9^ Then the project expanded to see the extent of radioactive strontium 90 in food pathways, looking at milk, soils and plants. Butts’ original USAEC work sparked his career as an international and UN FAO technical expert (Richards 2014: 203, 231–6).^10^

USAEC studies and scientists pioneered the field of radiation health safety. This involved much uncertainty and desperate means. They sometimes used whole skeletons and the bones of dead infants without the knowledge or consent of mourning loved ones. The secret methodology to acquire bodies, fetuses and body parts was not disclosed publicly (nor declassified until 1995, but in a 1996 interview, geochemist J. Laurence Kulp said only cadavers donated to medical schools were used). The USAEC released some of the Project Sunshine findings in 1956 to reassure the public that the amount of radioactive strontium, which displaces calcium to cause bone cancers and other harms and disorders, was trivial (Folkers 2021; Hamblin 2013:101–109; RAND 1953: 51).^11^

During the early 1950s, USAEC publicly voiced confidence in their frameworks for understanding radiation safety. Privately, controversy proliferated within scientific communities. Studies of the USAEC history show that organizational deviance with heuristic assumptions magnified confidence in radiation safety (Rice 2015). For example, the USAEC reported to the US Congress “all the harms from uranium were already understood” and therefore, such studies should be “curtailed” to focus instead on plutonium by 1950. “Partial evidence” already suggested that plutonium may be “less toxic than previously estimated.” Hopes were high that “work with plutonium in laboratories and plants could be simplified with substantial economies both in building facilities and in operating them” (USAEC 1951: 17). But radiation safety standards were contested at this time even within the radiation expert community on several criteria, including the lack of attention to genetic mutation. This can be seen in the tripartite conferences from 1949 to 1953, even though the participants included only USAEC invited Canadian, US and UK experts. There was much less certainty than the USAEC conveyed in their public statements for permissible doses. Regulations were not fully disseminated to the general public until 1954 (Richards 2014: 256; Stannard 1988: 1398).^12^

USAEC hopes that radiation could be rendered safe acted to obscure existing doubts. Safety was understood as essential, no matter how difficult to guarantee. Merril Eisenbud, Director of the USAEC’s New York HASL, who developed methods for Project Sunshine, later explained, “… if there is no threshold, there is no such thing as a safe dose. This made it necessary to pose a new question. How safe is safe enough?” (Eisenbud 1990: 221–2). In this quest for “safe enough,” optimism directed much of the work. In the early 1950s, some felt it was only a matter of time until scientists found a radiation antidote after injecting radioisotopes into patients or using a chelating agent to remove inhaled radionuclides, especially at Hanford Nuclear Reservation, a site of plutonium manufacture (USAEC 1951: 10, 17, 20–23; Stannard 1990: 1519, 1580–1).

## WHO’s Human Right to Health

USAEC safety science and Butts’ work contrasted in the same time period with Dr. Brock Chisholm’s idea of a human right to health. Chisholm shaped the very foundations of WHO (Baker 1950; Staples 2006: 143–7). The jurisdictional rivalries over control of radiation among the UN agencies reveal competing logics of radiation safety. While the USAEC conceptualized radiation regulations more as a collective roll of the dice with low probability and risk, WHO, with its medical individual emphasis, saw protection from radiation as a type of right to health.

Chisholm, the first Secretary General of the WHO, was a veteran of the trenches in World War I. He became a leading proponent of mental and emotional health for veterans and then led the Canadian health services before becoming an influential member of the 1946 UN preparatory committee that organized the WHO. A 1950 news article described the WHO mission as “revolutionary … because it defines health as ‘a state of complete physical, mental and social well-being and not merely the absence of disease or infirmity’”, and recognized “such a state of health” to be “one of the fundamental rights of every human being” essential “to the attainment of peace and security” (Baker 1950; Farley 2008). Chisholm’s vision of health as a human right inspired and animated WHO medical professionals (Staples 2006: 144–6).

Medical thinking at WHO prioritized the metaphorical patient as the focus. Chisholm conceptualized war and radiation harm in conjunction with his ideas of human rights to mental, physical, and public health. The use of nuclear and biological weapons and their threats on individual to social levels deeply troubled Chisholm. He said in 1947, “man’s own scientific knowledge” had become the greatest menace “capable of destroying the race.” For him, global levels of health, security and peace were inseparable from each human’s holistic wellbeing (Farley 2008: 3, 6, 190, 196–7; *New York Times *1971: 34).

With so much of the actual data of USAEC science off-limits as classified, requests for independent radiation research intensified in the late 1940s. However, this time period is marked by the hysteria of the Red Scare that pilloried those concerned about fallout or nuclear war as unpatriotic to western democracy. This upheaval accelerated in 1949 with the first Soviet nuclear weapon test and the victory of Mao Zedong in China. By the early 1950s the UN as a whole pivoted away from organizing actual scientific lab research “especially for public health” (as originally intended in the 1946 UN resolution) to fieldwork and technical assistance. America, the primary funder of WHO’s technical assistance projects, dismissed WHO’s concerns about harmful fallout from nuclear weapons tests as a political controversy that was irrelevant to the purposes of WHO. The USAEC dealt similarly with doubts inside the US among the public and from non USAEC scientists like chemist Linus Pauling (Siddiqi 1995: 13; Staples 2006; Wang 1999, 2002). Its orientation to the regulation of radiation questions favored economic development over health on a global scale.^13^

WHO had sincere commitments to protecting global public health (Farley 2008; de Chadarevian 2015: 359–74; Staples 2006: 145–53). This perhaps motivated international radiation experts to turn to WHO for impartial leadership on radiation research as early as 1950. The 1950 Sixth International Congress on Radiology recommended WHO address radiation protection as a global health issue. Despite USAEC blanket reassurances of the safety of fallout, rising radiation levels in the environment from US and Soviet thermonuclear weapons tests became impossible to hide. By February of 1953 Merril Eisenbud’s paper with J.H. Harley, “Radioactive Dust from Nuclear Detonations” was printed in *Science*. Eisenbud, Director of the USAEC’s Health and Safety Laboratory (HASL) acknowledged earlier estimates made of the amount of “safe” Sr-90 in the environment had already been exceeded. The paper’s findings were at “the lower end” of secret Project Gabriel’s projections of a global tolerance dose. Their 1953 paper included HASL’s literal numerical measures of radioactivity in maps and tables but concluded there was still no threat. Cumulative harms, they said, were “minute” when compared to lifetime doses of natural radioactivity from uranium and cosmic rays (Eisenbud & Harley 1953; Higuchi 2020: 16–60, 112, 138–9; Eisenbud 1990: 69–71, 77–79; Creager 2013: 157–169).

The request for WHO leadership was reiterated at the International Commission on Radiological Protection (ICRP) meeting in July of 1953. As a result, Austria formally requested WHO establish global radiation exposure regulations for workers and the public that year. On December 8, 1953, US President Dwight D. Eisenhower delivered his “Atoms for Peace” speech at the UN. An expansion of peaceful uses, already accelerating along with nuclear weapons tests, now posed additional unknowns. A follow up WHO report about radiation in January of 1954 concluded a “supranational organization” like WHO should make regulations “to protect people against radiations” as a whole population, including those “living in the neighborhood of an installation” with legally binding measures for all Member States.^14^

The WHO, with its medical and metaphysical roots, cared about individual biological humans. By the end of January, the WHO Executive Board debated issues of jurisdiction. Article 21 of the WHO Constitution limited WHO’s regulatory rulemaking to five major areas: disease; nomenclature; diagnostic practices; safety for products; and advertising and labeling. Some on the Executive Board felt WHO could only regulate in those five areas. US Rapporteur Dr. H. Hyde and Dr. C. van den Berg (Belgium) strongly opposed the proposal to study and regulate radiation as a global overreach. While such radiation regulations may be outside of the mandate of the WHO Constitution’s Article 21, Brazilian Marcolino Gomes Candau, the second WHO Secretary General from 1953 to 1973, argued WHO ought to act in accordance with other articles. Two other articles in the Constitution allowed WHO to make conventions or agreements among Member Nations “with respect to any matter within the competence of the Organization.” Candau felt WHO was the most competent in the public health sphere. He recommended WHO craft a convention, agreement or recommendation to Member States.^15^

Candau, like Chisholm, was deeply committed to protecting health. He served during a twenty-year period of decolonization when WHO membership increased by fifty-seven countries. All but three established national health systems with WHO’s help (WHO 2021; Staples 2006). Newly decolonialized states sometimes collectively organized against racism, fallout, and nuclear weapons (Intondi 2019). Some leaders made demands for access to non-weapons related nuclear technologies. In order to achieve rapid modernization, more practical investments to end poverty were often foreclosed (Phalkey 2013: 263–74, 290–1; Abraham 2018: 4, 5). A UNESCO pamphlet “Nuclear Energy and its Uses in Peace” enticed these future sovereign countries: “The stream of electrons that will gush from the power stations could become the lifeblood of underprivileged peoples” (Wendt 1955: 72). Areas of study delineated to WHO were listed in this same pamphlet. WHO’s ongoing projects included health physics training, addresssing health problems near reactors and waste disposal, the collection and distribution of information on radiation health, the standardization of units and codes of practice, and research coordination related to health aspects of radiation (Wendt 1955: 68–9).^16^ It seemed WHO would be the right agency to establish and coordinate radiation regulations, especially among emerging national public health agencies.

The USAEC response to the Lucky Dragon incident on March 1, 1954 derailed WHO’s efforts. The US Bravo thermonuclear test explosion on the Marshall Islands’ Bikini Atoll was a 1,000 times more powerful than the Hiroshima bomb. Radioactive fallout caused severe radiation sickness, even deaths, as it contaminated Marshall Islanders, Japanese fishermen at sea, and US servicemembers. US denials amid obvious and documented harm culminated in worldwide protests against nuclear weapons and tests. USAEC expertise in radiation safety contributed to the US ability to minimize controversy over the blast within the UN. International law and the UN trusteeship also restricted avenues of redress and protection for individuals to prevent harm caused by corporations and nations (Anghie 2010; Mitchell 2017: 215; Higuchi 2020: 41–87).

The USAEC quickly dispatched Merril Eisenbud to contain the damage within Japan as part of the US public relations response. He briefed the American members of the Tokyo press corps in a two hour “tutorial” on radiation and fallout and set up joint scientific meetings. But he could not prevent some Japanese scientists from independently studying fallout, or stop their “inflammatory statements” (Eisenbud 1990:98–9). The Japan Fishery organized research on the vessel *Lucky Dragon *to survey the radioactivity in the ocean from May until July. When these scientists returned, their studies showed nuclear pollution to be hundreds of times more severe, long lasting and vast than the USAEC had told the public. Radioactivity in the ocean and in bones had increased between 10 and 100 times in the last two years due to the tests. Hideki Yukawa, Yasushi Nishiwaki, and other scientists with the Science Council of Japan pointed out flaws in USAEC’s working model of radiation harm. Many Japanese scientists did not see evidence of a threshold below which there was no harm, encouraging scientists like Linus Pauling to ask more questions (Hamblin & Richards 2015).

By the summer of 1955 delineation of nuclear responsibilities among various UN agencies was in motion. Dag Hammarskjold, UN Secretary General, instructed Luther Evans, Director General of UNESCO, that each agency in the UN needed to “have on the table” their present and planned future atomic activities.^17^ By 1956, the ICRP had formally affiliated with WHO and WHO even inquired how their recommendations were decided that year (Clarke & Valentin 2009: 80, 92). It seemed WHO was in charge of radiation safety oversight.

The USAEC eventually coopted WHO’s role. US scientists like Eisenbud aided in many of the negotiations and diplomatic maneuvers that accompanied the US financial investment in the creation of the IAEA, an agency alluded to in Eisenhower’s Atoms for Peace speech (Roehrlich 2016). Eisenbud helped envision aspects of the IAEA as a member of the Preparatory Commission. Eisenbud sparked ideas for the first peaceful uses conference in Vienna, and helped initiate the UN Scientific Committee on the Effects of Atomic Radiation. All these supposed independent groups, however, effectively outmaneuvered WHO leadership and blocked Japanese hopes for unbiased UN research based on their interpretation of radiation harm (Eisenbud 1990: 85–97, 116–128). Despite the USAEC’s genesis of the agency as a part of Atoms for Peace, increasing fears and anxiety intensified the motivation for the IAEA (Krige 2006).

The UN’s initial 1946 resolution to organize scientific research for health was lost in the shift to technical assistance and development, which favored western nations and companies. They gained access to raw materials like uranium (Hill 1966: 112, 118; Staples 2006: 99; Hecht 2006, 2012, 2013). Experts and aid were often bound to tangible sales or investments, like General Atomics’ research reactors and mining ventures (Joubin & Smyth 1986: 394–5; Sharp 1952: 1–48).^18^ These programs could later be unexpectantly expensive for the IAEA and the recipient, and sometimes, as Mateos and Suárez-Díaz show for Mexico, certain nuclear technology could prove be a mismatch for the actual needs of the country (Mateos & Suárez-Díaz 2019: 361; 2020).

On the ground, UN technical experts instituted programs of nuclear education, science, and power for rapid development to improve living conditions and repair inequities (Joubin & Smyth 1986: 366–468; Alexander 1966). Scientists like Eisenbud and Butts had a genuine commitment to radiation safety. The allure of nuclear technology mixed with national security concerns, censorship, surveillance, patronage and uncertainty discouraged dedicated scientists from sometimes even recognizing questions to ask about radiation harm (Creager 2013; Nolan 2020; Higuchi 2020; Brown 2013). If they did ask, there could be a high price to pay. Even Eisenbud’s research to protect uranium miners proved futile (Proctor 1995).

It was UN experts, including geologists, who devotedly explored the nuclear potential and possibilities of each locale. They assessed the fitness for nuclear and health physics programs, research reactors, and prospects for uranium (Fischer 1997: 3, 331–52, 388). One such UN and IAEA expert, Canadian Franc R. Joubin, was a millionaire uranium prospector who had worked for Rio Tinto. Joubin served as a UN Technical Expert for thirteen years in countries ranging from Somalia to Haiti to the USSR, including with the IAEA in Pakistan (Joubin & Smyth 1986: 417–18, 453). He described himself as a “technical missionary,” as if he carried a nuclear gospel.

Such experts expanded power with their translocal nuclear knowledge. Butts moved from an ordinary agricultural college town to abroad and back while he lowered international trade barriers. He traveled for the USAEC and the FAO’s atomic branch (Fischer 1997: 373). Butts promoted Atoms for Peace during three assignments in Europe and one in the Middle East working in 19 cities to develop 11 research centers. He served while Chair of Agricultural Chemistry, expanding his department by five times. Butts secured USAEC patronage of at least “a half a million dollars” for radioisotope programs, studies and graduate students before he died while working on a joint UNICEF, WHO, and FAO children’s milk nutrition project in India (Richards 2014: 237–40). He was eulogized as having died in the line of duty for “the undernourished, the sick, the economically depressed and most of all, for those who had grasped a new vision for a better life.”^19^

For most scientists, using radiation was embraced as a useful research tool and a way to improve lives; it was not seen as irreversible contamination or genetic harm, or a violation of a right to health. Butts wrote in 1956 “too many people think of the Commission only in terms of bombs” but the USAEC was “the single most important force in applying nuclear energy and its products to biological and agricultural problems.”^20^ The Oregon Agricultural College’s Oceanography program’s studies of fallout in the Pacific demonstrate this (Stannard 1988: 794). They used USAEC and IAEA connections and grants for equipment from ships to computers by the early 1960s. Pollution from Hanford’s plutonium production for nuclear weapons, transported via the Columbia River to the sea, acted as a natural radioisotope tracer in order to research “plankton to whales” (Osterberg 1964: 243; Knapp 1974). Scientists like Kulp discovered strontium 90 could be a tracer to understand how calcium “turned over” in bones. He detected carbon 14 in 1952 from tests in Nevada on the US east coast and later, instigated the study of strontium 90 levels with Project Sunshine. In the mid 1950s, he and many scientists doubted such minuscule global fallout (if kept under their estimated threshold) carried more harm than a failure to test nuclear weapons to prepare for a possible Soviet attack.^21^

As the nuclear infrastructure worldwide multiplied, US public trust unraveled. In 1955, only seventeen percent of Americans even knew what fallout was. Two years later, fifty two percent felt fallout was dangerous (Walker 2000: 22). US Congressional Hearings (1957) on radiation effects intended to reassure the public backfired. Linus Pauling, along with actors from civil society and other scientists, protested nuclear weapons and tests, framing nuclear weapons tests, threats and pollution as intergenerational violations of human rights. Pauling also felt it was illogical to equate a threshold of safety with naturally occurring radiation from uranium and cosmic rays. This background radiation could be harmful by causing spontaneous abortions, birth defects, cancers, diseases, decreased immunity, shortened life span, genetic effects and intergenerational harms. Exposures were cumulative and not discrete events, especially if internal. Hot particles could act like bullets to break bonds to dispel the sense of radiation ever being “low” level or safe at any dose (Pauling 1983 [1958]; Richards 2013, 2014). By 1959 there was a “full scale radiation scare” with no denying that fallout contamination could be much more dangerous than supposed (Hacker 1994:198; Walker 2000: 22–3; Creager 2013: 173–77; US Congressional Hearing 1957: 16).

The USAEC could not necessarily prepare for nuclear war while protecting the public against radiation, and the tension between these aims put the entire nuclear project at risk. To rebuild trust, US President Eisenhower created a new agency in 1959, the Federal Radiation Council (FRC). Thresholds that Willard Libby (who directed Project Sunshine and solidified the motif of the threshold with measures of natural background radiation in his 1955 paper) insisted were more than safe enough in 1957 Congressional hearings were adjusted to be one-third more protective in 1959 (Richards 2014: 130–8; Libby 1955).

While the outcry caused radiation safety responsibilities to be bureaucratically transferred out of USAEC jurisdiction, the USAEC remained in control. Much of the work stayed in their hands, including interagency radiobiological health committees concerned with radioactive materials, biomedical effects of nuclear weapons tests, radiological emergency response and monitoring teams.^22^ The FRC contracted with the National Academy of Science and the National Commission on Radiological Protection for a review of “the biological and physiological models used by the FRC in developing its guidance for strontium 89, strontium 90, and cesium 137.” The more protective FRC threshold was found to be “well within the levels of exposure acceptable for a lifetime.” To grant the “maximum margin of safety, the upper limits … were related to the lowest possible level … that nuclear industrial technology could be developed” (Tompkins 1969: 500–1). The reviews did not seem to question the logic of the threshold or how the original data and numbers were determined (such as methods like Butt’s mathematical reconciliation of inconsistent results for Project Sunshine). The FRC solidified the USAEC value judgments of risk vs. benefit as an enduring philosophy of radiation protection (Walker & Mazuzan 1984: 261–75). Internationally, radiation oversight moved from the censured USAEC not to WHO, but instead to the IAEA by 1959. Why?

## 1954–64: WHO is in Charge of Public Health?

Four years before 1959, in 1955, Candau reported on WHO’s progress on research into harm from radiation to the UN and the World Health Assembly. He explained WHO was looking into genetics and somatic effects in 1955 “since the ambient radiation level is bound” to increase “by artificial means.” Candau wanted to know what effects “intensification of background radiation will have on mankind as soon as a large proportion of the world’s population is exposed to it.”^23^ While this focus might seem antithetical to the promotional USAEC views, the actual trainings and meetings of WHO were little threat to the motif of a safe threshold.^24^ Even the first WHO sponsored international training for health physics in 1955 held in Stockholm was a collaboration with USAEC.^25^

While Candau wanted more consideration of public health, the USAEC experts dominated the actual work. For example, James V. Neel, who studied fallout and formed the Atomic Bomb Casualty Commission (ABCC), chaired a 1959 WHO study of high natural radiation effects. The group included John Bugher (a USAEC Division of Biology and Medicine Director) who worked with Butts on Project Sunshine. Neel’s group was reliant on data from Project Sunshine. His group bolstered foundational arguments and estimates of the original calculation of naturally occurring background radiation, used to determine thresholds and how much radiation would be too much. These numbers were not reassessed, even though Project Sunshine was used to assure the public about fallout safety.^26^ The WHO study reproduced the tables, data and conclusions from earlier studies, whose origins were far from disinterested.^27^ Data fit the description of “black boxed” facts, obscuring the choices and biases that constructed them.

WHO interests were shaped by the USAEC connected director of radiation genetics and radioisotopes, Lowry Dobson (at WHO from 1958 to 1967). Under Dobson, WHO became involved in genetics research in much broader terms than radiation, deflecting attention from nuclear harm. WHO later became involved in “primitive populations” genetics projects led by Neel, who bespoke the primacy of the ABCC study results as opposed to other ways of understanding radiation harm (Goldstein & Stawkowski, 2015; de Chadarevian 2014: 90–2; de Chadarevian 2015). However, even while many experts like Eisenbud, Butts, Bugher, and Neel were on hand to plan UN meetings, training, and studies, Candau framed research questions as ways to understand harms from exposure.

Candau reported in January of 1959 the WHO radiation research program had “developed during the last few years much as originally outlined in 1954/55.” WHO had collated national models for possible standardizing of radiation protection legislation since 1955. Candau noted “legislation in the radiation protection field is difficult” but WHO was “developing basic data for use in recommendations of maximum permissible exposure … and included genetic concerns along with public protection from nuclear waste.” WHO centered on the “health aspects of radiation” with an effort to develop the activities that would help countries build their own programs in radiation health to “direct the attention of experts in the field to some of the large questions, answers to which are basic to sound health planning.” WHO researched “public health aspects of atomic energy, radioactive waste disposal, radiation legislation, and radiobiology” and the “study of health problems related to radiation and the peaceful uses of atomic energy, such as the effects of radiation on human heredity.” Candau also expressed hope for future research to prevent and understand internal exposures using the “field of analytical methods for radioactivity” in ways similar to the joint WHO/FAO 1958 Expert Committee on Radiochemical Methods of Analysis.^28^ WHO investigated genetics and heredity with studies in 1956 and 1958 and looked into cell and tissue culturing and chromosomal analysis techniques to track harmful effects.^29^ In Candau’s eyes, WHO remained ultimately responsible for protecting public health related to possible internal, cumulative, genetic, and environmental radiation contamination.

WHO was in the midst of “basic data collection,” collating standards, and creating regulations when USAEC’s chair John McCone outlined six tasks for the IAEA. Acting as a delegate at the IAEA’s second General Conference in 1958, McCone asked the IAEA to institute global radiation safety standards while solving “the third-party liability problem.” The US planned to finance research projects carried out in member countries under IAEA contracts.^30^ The US wanted their research questions and technology promoted under the aegis of IAEA using public private partnerships with uninsurable risks.

The same year in which oversight of radiation safety was taken from the USAEC and given to the FRC on paper, the IAEA increased US reach. During disputes with the IAEA in the spring of 1959 over how to account for radiation safety, and with what types of research, WHO signed an official Memo of Understanding (MOU) with the IAEA. The MOU assigned the IAEA a “primary” role per nuclear science. WHO agreed to communicate with IAEA before proceeding on any projects outside the realm of medical uses that concerned radiation. As subordinate agencies, WHO and FAO were both obliged to comply. The IAEA circumscribed radiation regulation similar to USAEC methods, making conflicts look like a consensus to increase their control over regulation and other agencies (Kuchinskaya 2014: 117; Boudia 2007; Hecht 2013; Hamblin 2009a: 25; 2021: 123–30). The ICRP formally affiliated that year with the IAEA, three years after it had done so with WHO (Clarke & Valentin 2009: 80). On May 11, 1959, USAEC entered into a Section 123 bilateral agreement with the IAEA.^31^ 1959 marks the shift from USAEC to IAEA as the locus for nuclear expansion using the USAEC-created radiation regulation risk model.

USAEC experts served in many nations under UN agency technical assistance programs in UNESCO, FAO and WHO, followed later by the IAEA. This returned benefits to the US as the gift-giver (Rentetzi 2021). The IAEA’s efforts to meet requests for technical experts and to standardize national programs of radiation health safety were initially slow. By the early 1960s their program became much more organized. Thirty IAEA technical nuclear and raw materials experts were sent to twenty countries after 1961, including six health physics experts. These IAEA experts mostly worked through national atomic agencies. From 1961 to 1963 IAEA health physics experts went to the United Arab Republic (Egypt), Iraq, Israel, Thailand, Ghana, Iran, Greece and the Philippines.^32^

What did these health physics radiation protection experts do? Hanson Blatz had studied uranium harms and fallout with Eisenbud at the USAEC’s Health and Safety Laboratory in the 1950s. Like many IAEA and WHO health physics experts, Blatz worked on the problems of inconsistent dosimetry, helping to calibrate instruments and implement legislation for the host country.^33^ Blatz (assigned with WHO) established the radiation health safety program in Thailand with IAEA technical expert R.A. Borthwick, a New Zealander, in 1963. Borthwick had served as a UN technical expert in 1962 during the construction of the first Philippine research reactor. He instituted environmental monitoring near the reactor, established a nationwide radiation badge service to include hospitals, and created safety manuals. Borthwick also redefined the terminology in legislation that concerned maximum permissible dose for the Philippines. This made the rules standard, though in some cases also less stringent. He was involved with legislation and regulations of radiation as a WHO expert as well, serving to align radiation safety in Pakistan and Nigeria with western norms.^34^ WHO technical experts seemed to embrace their role as responsible for instituting IAEA standardized radiation regulations in national legislatures and medical equipment safety.

Some USAEC experts conceptualized harm in ways that deflected WHO opportunities to question safety. For example, USAEC scientist J. Newell Stannard, key to the experimental origins of radiation safety standards, organized and led a 1961 WHO/IAEA health physics training in Chiba City, Japan (Stannard 2003: 205). Another example is Austin Brues, Director of the USAEC Division of Biological and Medical Research. He assembled a 1961 WHO meeting “Radiation Hazards in Perspective.” Although it was an international group, the USAEC perspective dominated. The scientists admitted that decreased life span and accelerated aging had been found in animals exposed to radiation. Yet, they concluded this must be weighed against the increase in life span due to nuclear medicine. They advised public health education should dispel “public anxiety and mysticism” around radiation to rank it properly against other more severe risks. They argued attention to radiation had caused neglect of dangerous chemical pollutants, like toxins and smoking, which should be studied instead.^35^

Yet, Candau still resisted relinquishing WHO’s role in radiation, nuclear technology and health entirely to the IAEA. Despite the 1959 MOU, correspondence from 1963 to 1964 casts doubt Candau ever gave up on his hopes WHO might contribute to better radiation health protection.^36^ IAEA standards focused predominantly on protection of lab workers. WHO’s focus by this time was narrowed to only medical devices, leaving no real global mechanism for oversight to prevent uranium, intergenerational, public or environmental contamination.^37^

In spring and summer of 1963, Candau asserted the defense of public health required WHO to consider “radioactive environmental pollutants” as part of its responsibility. The IAEA staff saw this as disruptive of their plans to convene a panel to “consider acceptable emergency doses to the public” in case of a nuclear power plant accident. IAEA staff argued WHO did not have the capability to do what the IAEA could do, but admitted that having WHO as a co-sponsor lent legitimacy to IAEA regulations, especially in hospital and medical settings.^38^ Complaints on both sides included UN meetings being planned without participation or consultation between agencies.

Differences in priorities and philosophy fueled the fight. Candau felt developing countries needed investments in basic sanitation to prevent disease, not nuclear medicine (Fischer 1997: 59, 212, 421). Candau chastised the IAEA at this time for intruding on other country’s health programs without concern for regional coordination or assessing actual needs. He identified IAEA budgeted health symposiums and meetings that ought to have been under the purview of WHO in Korea, Senegal, Tunisia, Nigeria and Pakistan. In response, IAEA staff feared they could lose almost $1.4 million dollars, a sixty percent loss of their Expanded Programme of Technical Assistance budget in 1964 due to WHO encroachment on their IAEA medical, health protection, agricultural, power, hydrology and training projects.^39^

Candau diplomatically appealed to IAEA Secretary General Sigvard Eklund to end this “undesirable program duplication.” The IAEA intended to recategorize medical facilities as “small nuclear establishments.” This could eliminate WHO’s control over radioisotope and medical health physics in hospitals and clinics. Developing countries, Candau argued, should not be subject to the same mistakes made by other countries decades before, when those promoting atomic energy were also responsible for public health. Candau was “perturbed” by the IAEA’s plan to convene the panel on public emergency doses. He wrote to Eklund: “It seems particularly inappropriate for basic recommendations on such matters as acceptable levels of radiation exposure of the public to be promulgated by an atomic energy agency.”^40^ Candau reiterated WHO was the proper party responsible for public health and should be setting the standards, even as he tried to cooperate on other matters with the IAEA.^41^ His effort to protect human health from radiological exposure had to be balanced against “depriving the world” of the benefits of nuclear power technology (Hamblin 2009b: 60–1).

Nuclear technologies that had been installed before resolving questions about health, resulted in the abandonment of Candau’s plans for “sound health planning.” Peaceful nuclear science and technology distracted from both weapons testing and contamination, while sometimes even creating a capacity for nuclear weapons proliferation (Leslie 2015). Krige & Sarkar (2018) argue in some cases technical assistance successfully deflected interest in making nuclear weapons. Either way, the USAEC buttressed the IAEA to be accepted as a watchdog and gatekeeper for science and peace (Rentetzi 2017; Roehrlich 2016; Mateos & Suárez-Díaz 2015).

## Making and Breaking Bonds

This inchoate story is a plea for more UN research to understand how the USAEC logic of radiation safety took root and traveled, and at what cost these research agendas were pursued. How might this story be different if the UN had organized research labs in 1946 “especially for public health”? USAEC rendering of radiation regulations to suit the production of nuclear weapons was integral to the spread of nuclear technology. The producers and users of the technology made the rules and created radiation experiments with grave consequences. Generously funded and seemingly certain studies forestalled other ways of knowing or perceiving harm as it cultivated bonds among scientists with expertise in the safe threshold model.

The USAEC operationalized and then shepherded one way of seeing radiation: levels of exposure below naturally occurring background radiation were held to be legally safe enough. USAEC scientists played a central role in rooting these viewpoints in place, in minds and in law. Much more could be learned about how science travels with UN technical experts. USAEC experts spread their view of radiation from national labs to college campuses to the UN to developing nations, and then back home again. The early involvement of experts like Butts, Brues, Bugher, Blatz, Stannard, Neel and Eisenbud in classified projects entrusted loyalty to the threshold model they helped make while they sought to find the safest levels according to this threshold concept. They instituted safety mechanisms where they could, but assuaged what they felt were often exaggerated fears of radiation for the rest of their lives.

Scientists’ embrace of the threshold model assumed and naturalized human exposure to ionizing radiation. Many scientists who were bound to the USAEC christened exposures as trivial if under these limits; ultimately, they held contamination to be worth the risks for the promise of unlimited energy and medicine. In doing so, their humanitarian impulse for a bright future shaped how exposures are seen as risks, but not as violations of rights to health.

IAEA and USAEC experts shared a modernist utopian ideology. Seeing as nations often do, the USAEC and IAEA accomplished goals of standardization using probabilities and statistics, invalidating those opposed with tactics of authority and coercion (Scott 1998: 1–8). Local to global bureaucratic and legislative UN mechanisms further bolstered the authority of the USAEC and IAEA. Experts obscured seeing radiation as a public harm to be protected against, at a time before jurisdiction and authority over radiation regulation were fully decided at the UN. A state centric view placed the desire to promote and develop nuclear technologies at the forefront, limiting other ways of seeing contamination. Seeing contamination as a risk and not as a danger kept the focus on how much exposure was allowed to sanction it. This produced high barriers for redress for exposed people and communities worldwide (Hu 2020; Mitchell 2017; Anghie 2010).

Despite the discrediting of the USAEC in 1959, their state centric model of “how safe is safe enough?” continues to hold primacy and patronage. American priorities and their influence in the UN circumscribed debates, relegating them to questions about just how much was safe enough. But safe enough for whom? A 5′7″, 154 pound, 20–30 year old Caucasian male with a western diet stands in as the reference for all ages and bodies (this basis leads to disproportionate harm to subsistence cultures, women and children, see Markstrom & Charley 2006; Olson 2019; Folkers 2021). The lack of equally funded independent research combined with tactics of control that were implemented in UN meetings and studies insulated the main logics and premise of the threshold against serious criticism.

IAEA aegis rapidly expanded the technology to developing countries. UN technical experts were essential to institute safety legislatively with regulations. Experts installed radiation practice and rules using the privileges of UN access to individual countries’ legislatures. This mirrored similar processes at US state legislatures. UN experts, without much notice or discussion, inserted the threshold model into emerging national regulatory and legal codes, in country after country (Richards 2014: 199–243).

This erased the brutal experimental origins of health physics. As of April 2021, 173 member states of the IAEA follow this risk model of legally allowable exposure limits (IAEA List 2022a). According to the IAEA, these standards “reflect an international consensus on what constitutes a high level of safety for protecting people and the environment from harmful effects of ionizing radiation” (IAEA Safety 2022b).

Chisholm, Candau, Pauling and others articulated a human right to health after Hiroshima and Nagasaki. They argued it was a fundamental right to live free without contamination or threats of nuclear war and pollution. Chisholm communicated this right to health as a type of intergenerational holism. He said in 1950, “The mature person needs to be responsible to all humanity; in relation to time, space and to all human beings” (Chisholm 1951 [1950]: 4). The IAEA made the nuclear order a reality despite such concerns, eclipsing WHO’s responsibility for public protection from harm as a human right to health.

Radiation contamination can break human bonds along ethical, genetic, mental, social, emotional, intergenerational, and spiritual dimensions (Markstrom & Charley 2006; Kurihara 1999; Shrader-Frechette 2013, 2017). The suffering and stories of radiation harmed communities and individuals can be as invisible as the radiation (Jacobs 2014; Fox 2014; Uranium Atlas 2020; Hamblin & Richards 2021). Imposing risks while failing to see, or consider, the harms is incommensurate with human rights regimes (Grover, Anand 2013; Nadesan 2013, 2019). It is Indigenous and other nuclear contaminated individuals and communities who are often leaders for human rights to health (Doulatram 2018; Grover 2021; Sonoda 2021). Their claims belong in debates about the reality of human rights (Moyn 2010; Cargas 2016; Niezen 2020). Interweaving UN, radiation, and human rights histories might chart a map for understanding these broken bonds.

## Footnotes

1 Records of the Atomic Energy Commission (AEC), RG 326, National Archives at College Park, Maryland (NARA II). “Quarterly Progress Report to the JCAE, July–Sept” 1958 p. 32, 53–64, accessed May 9, 2022 https://www.osti.gov/opennet/servlets/purl/16091912.pdf; “The International Atomic Energy Agency, by International Review Service, January, 1957” Ava Helen and Linus Pauling Papers, (AHLPP), Linus Pauling (LP) Peace, Box 3.018, Folder 8.6, Special Collections and Archives Research Center (SCARC), Oregon State University, Corvallis, Oregon; Rene Maheau to Gagliotti, December 21, 1958 and Cacciapuoti to Sievert November 19, 1958 620.992:539.16 Atomic Radiations (includes UN Scientific CT on the Effects of Atomic Radiation) Part II from 1.1.1957 AG 8 UNESCO Archives, Paris.

2 “Project Number 5 for Argentina” in Folder “Argentina Bergstorm L.I. October 1958–June 1959” Teaching of Science, Nuclear Physics, UNESCO Index of Field Mission Reports 1947–1968 AG 8, UNESCO Archives.

3 22(IIII) United Nations Research Laboratories, Resolution of 3 October 1946, E/233, p. 49–50, 1947.

4 “The Radiation Health Work of the WHO in Historical Perspective” u.d. p. 1, EB 33, 46 Annex 2, World Health Organization (WHO), O/320‑2, folder 1963–4, Box 04204, IAEA Archives, Vienna.

5 See DOE “Human Radiation Experiments” and “Advisory Committee on Human Radiation Experiments (ACHRE)” accessed May 9, 2022 https://www.osti.gov/opennet/ and “Spotlight on Human Radiation Experiments” accessed May 7, 2022 https://www.osti.gov/opennet/spotlight or https://www.osti.gov/opennet/spotlight.jsp; For the implications of just one study, see Lawrence, Pope A., Papers 1924–1983, MS C 539, Series III: Air Pollution Control Office, 1924–1979, Uranium Miner’s Study, Radiation and Uranium Miners, United States National Library of Medicine, Bethesda, MD.

6 Joseph S. Butts Papers (MSS Butts), SCARC; “Butts, Joseph S” Employment Records, SCARC.

7 Ibid; OSC News Bureau Photo, July 13, 1950, Gwil Evans Photographic Collection, 1930–1968 (P 082) SCARC Historical Images of Oregon State University, Science, 1951, accessed May 6, 2022 https://oregondigital.org/catalog/oregondigital:df739909b.

8 Records of the AEC in Series V. Box 8, RG 62 and RG 326, NARA II, and in the E. Dale Trout Papers, 1939–1983, SCARC, Corvallis, Oregon.

9 Quotation from Butts (AEC Division of Biology and Medicine) to N.S. MacDonald, May 24, 1954, “Interlaboratory Comparison of Analyses for Elemental Strontium” US DOE 326 US Atomic Energy, Division of Biology and Medicine, Box 3365 Folder 3, NARA, NNSA/NSO Nuclear Testing Archive, Accession Number NV0721134. See also H.H. Mitchell May 24, 1954, “Comparison of Analyses for Elemental Strontium” NNSA/NSO Nuclear Testing Archive, Accession Number NV0721133, DOE OpenNet; Interview of Dr. J. Laurence Kulp by Ron Doel on April 11 and 12, 1996, Niels Bohr Library & Archives, American Institute of Physics (AIP), College Park, MD, accessed May 4, 2022 https://www.aip.org/history-programs/niels-bohr-library/oral-histories/6932-1 and https://www.aip.org/history-programs/niels-bohr-library/oral-histories/6932-2.

10 “Meeting notes” and Queen to Weniger February 12, 1948, “Memorandum Meeting with Dr. Queen,” January 6, 1948, Strand President File, SCARC; G.W. Gleeson to Leighton Collins, October 14, 1958, RG 62, Series V, Box 8, File AEC Summer Institute, 1958–1968, NARA II; Butts to Price and Gilfillan August 22, 1952 “Butts, Joseph S” Employment Records 1 of 3, and Eulogy by either Paul Weswig or OAC President Strand, “In Memory of Joseph Shirley Butts” “Butts, Joseph S” Employment Records 2 of 3, and Pearson to Butts, August 16, 1954; “Rand Sunshine Project” 3, this page lists all participants in the Washington DC Sunshine Meeting January 9 and 10, 1954, File “US Atomic Energy Commission, Project Sunshine Reports Folder #1” MP 1997-0004 Miscellaneous Physics, American Institute of Physics, College Park, MD.

11 Interview of Dr. J. Laurence Kulp by Ron Doel on April 11 and 12, 1996, Niels Bohr Library & Archives, AIP), accessed May 4, 2022 https://www.aip.org/history-programs/niels-bohr-library/oral-histories/6932-1 and https://www.aip.org/history-programs/niels-bohr-library/oral-histories/6932-2; Memorandum: Update on Project Sunshine “Body Snatching”, 5 ACHRE June 9, 1995, br15d2.txt, see also br15d2a, br15d2b, br15d2c files which includes transcripts of a January 18, 1955 “Biophysics Conference” convened by the AEC Division of Biology and Medicine, George Washington University, National Security Archives, accessed May 10, 2022, https://nsarchive2.gwu.edu/radiation/dir/mstreet/commeet/meet15/brief15/tab_d/br15d2.txt. Other supporting ACHRE materials, can be viewed at https://nsarchive2.gwu.edu/radiation/, accessed May 9, 2022.

12 US Department of Commerce, “Permissible Doses from External Sources of Ionizing Radiation” September 24, 1954, 7.001.4 LP Peace AHLPP, SCARC.

13 “The Radiation Health Work of the WHO in Historical Perspective” u.d. p. 1, EB 33, 46 Annex 2, World Health Organization (Correspondence WHO) O/320‑2, folder 1963–4, Box 04204, IAEA Archives.

14 Dr. A.L. Bravo, 21 January 1954 “Briefing on Protection Against Radiations—Item I of the Supplemental Agenda” National Legislation Against Radiations folio A 14/288/2 Jacket 1 WHO Archives, Geneva. Dr. Zakowsky compiled the report. The ICRP is an international body of radiation equipment users, producers and scientists that advised on radiation protection since the late 1920s. How many other important organizations and individuals intersect with this story is for future research.

15 Constitution of World Health Organization, *Basic Documents*, 2006, accessed May 9, 2022 https://apps.who.int/gb/bd/pdf_files/BD_49th-en.pdf#page=6http://www.who.int/governance/eb/who_constitution_en.pdf; “Minutes of the Twenty-fourth Meeting 29 January 1954” 19–21, 15 March 1954, 19–21 EB13/Min/24 Rev.1 National Legislation Against Radiations folio A 14/288/2 Jacket 1, WHO Archives.

16 “The Radiation Health Work of the WHO in Historical Perspective” u.d. EB 33, 46 Annex 2, WHO-, O/320‑2, folder 1963–4, Box 04204, IAEA Archives.

17 Dag Hammarskjold to Dr. Evans, August 28, 1955, UNESCO AG 8 Secretariat Records, 1946—File 620.992:3 A 06 (44) “58” Expert Meeting on the Social and Moral Implications of the Peaceful Uses of Atomic Energy, France 1958 Part 1 up to 30/6/ UNESCO Archives.

18 Extensive records of nuclear commerce are found throughout USAEC files and records groups in NARA as nuclear programs on campuses and in other nations were being established; Dale Trout Papers, SCARC; “United States Policy Toward Dependent Territories” April 26, 1950 Foreign Relations of the United States, 1952–1954 Volume III, United Nations Affairs, Document 775 Department of State Committee files, lot 54 D 5, “Working Group on Colonial Problems” Paper Prepared by the Colonial Policy Review Sub-Committee of the Committee on Problems of Dependent Areas, accessed October 23, 2021, https://history.state.gov/historicaldocuments/frus1952-54v03/d775 and http://history.state.gov/historicaldocuments/frus1952-54v03/d778.

19 Morris Greene, Regional Administrative Officer FAO to Strand, July 26, 1960, Robert G. Swan to Norblad, May 15, 1961, “Butts, Joseph S” Employment Records, 2 of 3, SCARC.

20 “Butts, Joseph S” Employment Records, 3 of 3 “Isotopes in Agriculture” 1956, Butts Papers, SCARC.

21 Interview of Dr. J. Laurence Kulp by Ron Doel on April 11 and 12 1996, AIP, accessed May 4, 2022 https://www.aip.org/history-programs/niels-bohr-library/oral-histories/6932-1 and https://www.aip.org/history-programs/niels-bohr-library/oral-histories/6932-2.

22 Arthur S. Flemming to Robert Gray, u.d. November. The Federal Radiation Council May 5–March 24, 1960 and November 17, 1959, White House Office Cabinet Secretariat Records 1953–1960, Box 10, File “Radiation Council” Dwight D. Eisenhower Presidential Library Papers, Abilene, Kansas.

23 World Health Organization & Candau, Marcolino Gomes. (1956). The Work of WHO, 1955: Annual Report of the Director-General to the World Health Assembly and to the United Nations. World Health Organization, quote on page x, accessed May 9, 2022 https://apps.who.int/iris/handle/10665/85666.

24 Executive Board, 23. (1959). A brief survey of the WHO programmes in radiation and isotopes. World Health Organization. https://apps.who.int/iris/handle/10665/133167; World Health Organization & Candau, Marcolino Gomes. (1956). The work of WHO, 1955: Annual Report of the Director-General to the World Health Assembly and to the United Nations. World Health Organization, accessed May 9, 2022 https://apps.who.int/iris/handle/10665/85666.

25 World Health Organization & Candau, Marcolino Gomes. (1956). The Work of WHO, page x, accessed May 9, 2022 https://apps.who.int/iris/handle/10665/85666

26 WASH–406 (Rev) (Deleted Version) “Radiostrontium Fallout: Project Sunshine” by W.F. Libby, July 1956, Human Studies Project Team, Los Alamos National Laboratory, April 29, 1994 HSPT–REL–94–444.

27 First Report of the Expert Committee on Radiation, “Effect of Radiation on Human Heredity: Investigations of Areas of High Natural Radiation” *World Health Organization Technical Report Series* No. 166, (Geneva: WHO, 1959).

28 Executive Board, 23. (1959). A brief survey of the WHO programmes in radiation and isotopes. World Health Organization, accessed May 9, 2022 https://apps.who.int/iris/handle/10665/133167.

29 “The Radiation Health Work of the WHO in Historical Perspective” u.d. EB 33, 46 Annex 2, WHO), O/320‑2, folder 1963–4, Box 04204, IAEA Archives.

30 “Quarterly Progress Report to the JCAE, July–Sept” 1958 p. 32, 53–64 accessed May 9, 2022 https://www.osti.gov/opennet/servlets/purl/16091912.pdf

31 “Agreement Between the IAEA and WHO” (u.d. but 1959); “The Radiation Health Work of the WHO in Historical Perspective” u.d. EB 33, 46 Annex 2, WHO, O/320–2, folder 1963–4, Box 04204, IAEA Archives; The particular MOU document “Approved by the Twelfth World Health Assembly on 28 May 1959 in resolution WHA12.40” is preserved for the public in a wiki page, accessed May 4, 2022 https://en.wikisource.org/wiki/Agreement_between_the_World_Health_Organisation_and_the_International_Atomic_Energy_Agency.

32 IAEA, “Technical Assistance for Radiation Protection: Work of Some IAEA Experts in Health Physics,” 1963, 33–6, accessed May 3, 2022, https://www.iaea.org/sites/default/files/publications/magazines/bulletin/bull5-3/05305303336.pdf.

33 Hanson Blatz, “Assignment Report on the Development of Radiological Protection Services in Thailand, April 1963” SEA/Rad/13. 26.6. 63 Restricted Radiation-Thailand D63.1360, 2–7, AG 8 WHO Archives; IAEA, “Technical Assistance for Radiation Protection,” 33–6.

34 Borthwick to Larsson, May 21, 1964 and R. Lowry Dobson to Borthwick, June 27, 1960 A 14/288/2 Jacket 1 “National Legislation for Protection Against Radiations” AG 8 WHO Archives; IAEA, “Technical Assistance for Radiation Protection” 35–6; Blatz, “Assignment Report on the Development of Radiological Protection Services in Thailand,” 2–7, AG 8 WHO Archives.

35 WHO Technical Report Series No. 248 *Radiation Hazards in Perspective: Third Report of the Expert Committee on Radiation *quotation on 1, 34–5, 38 (Geneva: WHO 1962) WHO Archives.

36 “The Radiation Health Work of the WHO in Historical Perspective” u.d. EB 33, 46 Annex 2; Elkund to Candu, 16 June, 1964; Candau to Elkund, Nov. 3, 1964 and Sept. 3, 1964; P. Balligand to Director General “Interoffice Memorandum” 26 August 1963; “Urgent: DAV Fisher to all Deputy Directors General, WHO/IAEA Relations, 20 May 1963” 1, 2; Candau to Sigmund Elkland, 11 April 1963; “Notes from Talk with Dr. Candau 8 February 1963”; “WHO Meeting: Background Points,”; “Answers to WHO’s Arguments” 1; all documents in WHO, O/320–2, folder 1963–4, Box 04204, IAEA Archives.

37 S.V. Arnaldo to Director General, November 1, 1955, 3 and Director General to S.V. Arnaldo, November 2, 1955, file 620.992:539.16 “Atomic Radiations (includes UN Scientific CT on the Effects of Atomic Radiation)” Part 1 up to 31.XII.56 AG 8 UNESCO Archives. Both WHO and IAEA files during the 50s and early 60s concerning the other show these ongoing tensions at both of their archives in Vienna and Geneva.

38 P. Balligand to Director General “Interoffice Memorandum” 26 August 1963; “Urgent: DAV Fisher to all Deputy Directors General, WHO/IAEA Relations, 20 May 1963” 1, 2; Candau to Sigmund Elkland, 11 April 1963; “Notes from Talk with Dr. Candau 8 February 1963”; “WHO Meeting: Background Points,”; “Answers to WHO’s Arguments” 1; all documents in WHO, O/320–2, folder 1963–4, Box 04204, IAEA Archives.

39 “WHO Meeting: Background Points,”; “Answers to WHO’s Arguments” 1, WHO, O/320–2, folder 1963–4, Box 04204, IAEA Archives; World Health Organization (WHO/AD/1-93.1959) 1959, Radiation Legislation, National Laws on Radiological Protection (WHO/AD/9.59) Radiations 1:59.358, WHO Archives.

40 “IAEA/WHO Co-ordination,” Candau to IAEA Director General, April 30, 1963, Annex III, EB-33/46, WHO, O/320–2, folder 1963–4, Box 04204, IAEA Archives. Quotations pages 1 and 2.

41 Candau to Eklund November 3, 1964 and Candau to Eklund November 12, 1964, WHO, O/320–2, folder 1963–4, Box 04204, IAEA Archives.
